# A systematic literature review and narrative synthesis on the risks of medical discharge letters for patients’ safety

**DOI:** 10.1186/s12913-019-3989-1

**Published:** 2019-03-12

**Authors:** Christine Maria Schwarz, Magdalena Hoffmann, Petra Schwarz, Lars-Peter Kamolz, Gernot Brunner, Gerald Sendlhofer

**Affiliations:** 1 Research Unit for Safety in Health, c/o Division of Plastic, Aesthetic and Reconstructive Surgery, Department of Surgery, Medical University of Graz, Graz, Austria; 2 Executive Department for Quality and Risk Management, University Hospital Graz, Auenbruggerplatz 1/3, 8036, Graz, Austria; 3 Carinthia University of Applied Science, Feldkirchen, Austria

**Keywords:** Discharge letter, Discharge summary, Risk, Patient safety, Hospital discharge, Systematic review

## Abstract

**Background:**

The medical discharge letter is an important communication tool between hospitals and other healthcare providers. Despite its high status, it often does not meet the desired requirements in everyday clinical practice. Occurring risks create barriers for patients and doctors. This present review summarizes risks of the medical discharge letter.

**Methods:**

The research question was answered with a systematic literature research and results were summarized narratively. A literature search in the databases PubMed and Cochrane Library for Studies between January 2008 and May 2018 was performed. Two authors reviewed the full texts of potentially relevant studies to determine eligibility for inclusion. Literature on possible risks associated with the medical discharge letter was discussed.

**Results:**

In total, 29 studies were included in this review. The major identified risk factors are the delayed sending of the discharge letter to doctors for further treatments, unintelligible (not patient-centered) medical discharge letters, low quality of the discharge letter, and lack of information as well as absence of training in writing medical discharge letters during medical education.

**Conclusions:**

Multiple risks factors are associated with the medical discharge letter. There is a need for further research to improve the quality of the medical discharge letter to minimize risks and increase patients’ safety.

## Background

The medical discharge letter is an important communication medium between hospitals and general practitioners (GPs) and an important legal document for any queries from insurance carriers, health insurance companies, and lawyers [1]. Furthermore, the medical discharge letter is an important document for the patient itself.

A timely transmission of the letter, a clear documentation of findings, an adequate assessment of the disease as well as understandable recommendations for follow-up care are essential aspects of the medical discharge letter [2]. Despite this importance, medical discharge letters are often insufficient in content and form [3]. It is also remarkable that writing of medical discharge letters is often not a particular subject in the medical education [4]. Nevertheless, the medical discharge letter is an important medical document as it contains a summary of the patient’s hospital admission, diagnosis and therapy, information on the patient’s medical history, medication, as well as recommendations for continuity of treatment. A rapid transmission of essential findings and recommendations for further treatment is of great interest to the patient (as well as relatives and other persons that are involved in the patients’ caring) and their current and future physicians. In most acute care hospitals, patients receive a preliminary medical discharge letter (short discharge letter) with diagnoses and treatment recommendations on the day of discharge [5]. Unfortunately, though, the full hospital medical discharge letter, which is often received with great delay, is an area of constant conflict between GPs and hospital doctors [1]. Thus the medical discharge letter does not only represent a feature of process and outcome quality of a clinic, but also influences confidence building and binding of resident physicians to the hospital [6].

Beside the transmission of patients’ findings from physician to physician, the delivery of essential information to the patient is an underestimated purpose of the medical discharge letter [7]. The medical discharge letter is often characterized by a complex medical language that is often not understood by the patients. In recent years, patient-centered/patient-directed medical discharge letters are more in discussion [8]. Thus, the medical discharge letter points out risks for patients and physicians while simultaneously creating barriers between them.

### Aim

A systematic review of the literature was undertaken to identify patient safety risks associated with the medical discharge letter.

## Methods

### Search strategy

A systematic literature search was conducted using the electronic databases PubMed and Cochrane Database. Additionally, we scanned the reference lists of selected articles (snowballing). The following search terms were used: “discharge summary AND risks”, “discharge summary AND risks AND patient safety” and “discharge letter AND risks” and “discharge letter AND risks AND patient safety”. We reviewed relevant titles and abstracts on English and German literature published between January 2008 and May 2018 and started the search at the beginning of February 2018 and finished it at the end of May 2018.

### Eligibility criteria

In this systematic review, articles were included if the title and/or abstract indicated the report of results of original research studies using quantitative, qualitative, or mixed method approaches. Studies in paediatric settings or studies that do not handle possible risks of the medical discharge letter were excluded, as well as reports, commentaries and letters. Electronic citations, including available abstracts of all articles retrieved from the search, were screened by two authors to select reports for full-text review. Duplicates were removed from the initial search. Nevertheless, during the search of articles the selection, publication as well as language bias must be considered. Thereafter, full-texts of potentially relevant studies were reviewed to determine eligibility for inclusion. In the following Table 1 inclusion and exclusion criteria for the studies are listed. Afterwards, key outcomes and main results were summarized. Differences were resolved by consensus. Finally, a narrative synthesis of studies meeting the inclusion criteria was conducted. Reference management software MENDELEY (Version 1.19.3) was used to organise and store the literature.

**Table 1:** Criteria for inclusion and exclusion of studies

Criteria	Inclusion	Exclusion
Time period	January 2008– May 2018	Before 2008
Language	German, English	Other languages
Setting	Studies with adults Patients at discharge to referring physicians or GPs	No studies in pediatric settings Patient transfer within the hospital or to another hospital, or patient hand-over situations
Type of studies	Primary studies	Reports, commentaries, letters
Aim: to identify risks of the medical discharge letter	Literature points out possible risks or challenges of the medical discharge letter	Literature does not cover challenges or risks in terms of the medical discharge letter

### Data extraction

The data extraction in form of a table was used to summarize study results. The two authors extracted the data relating to author, country, year, study design, and outcome measure as well as potential risk factors to patient safety directly into a pre-formatted data collection form. After data extraction, the literature was discussed and synthesized into themes. The evaluation of the single studies was done using checklists [STROBE (combined) and the Cochrane Data collection form for intervention reviews (RCTs and non-RCTs)]. Meta-analysis was not considered appropriate for this body of literature because of the wide variability of studies in relation to research design, study population, types of interventions and outcomes.

### Synthesis

Then a narrative synthesis was performed to synthesize the findings of the different studies. Because of the range of very different studies that were included in this systematic review, we have decided that a narrative synthesis constitutes the best instrument to synthesise the findings of the studies. First, a preliminary synthesis was undertaken in form of a thematic analysis involving searching of studies, listing and presenting results in tabular form. Then the results were discussed again and structured into themes. Afterwards, summarizing of included studies in a narrative synthesis within a framework was performed by one author.

This framework consisted of the following factors: the individuals and the environment involved in the studies (doctors, hospitals), the tools and technology (such as discharge letter delivery systems), the content of the medical discharge letter (such as missing content, quality of content), the accuracy and timeliness of transfer. These themes were discussed in relation to potential risks for patient’s safety. All articles that were included in this review were published before. The framework of this study was chosen following a previously published systematic review dealing with patient risks associated with telecare [9].

## Results

**Fig. 1:**
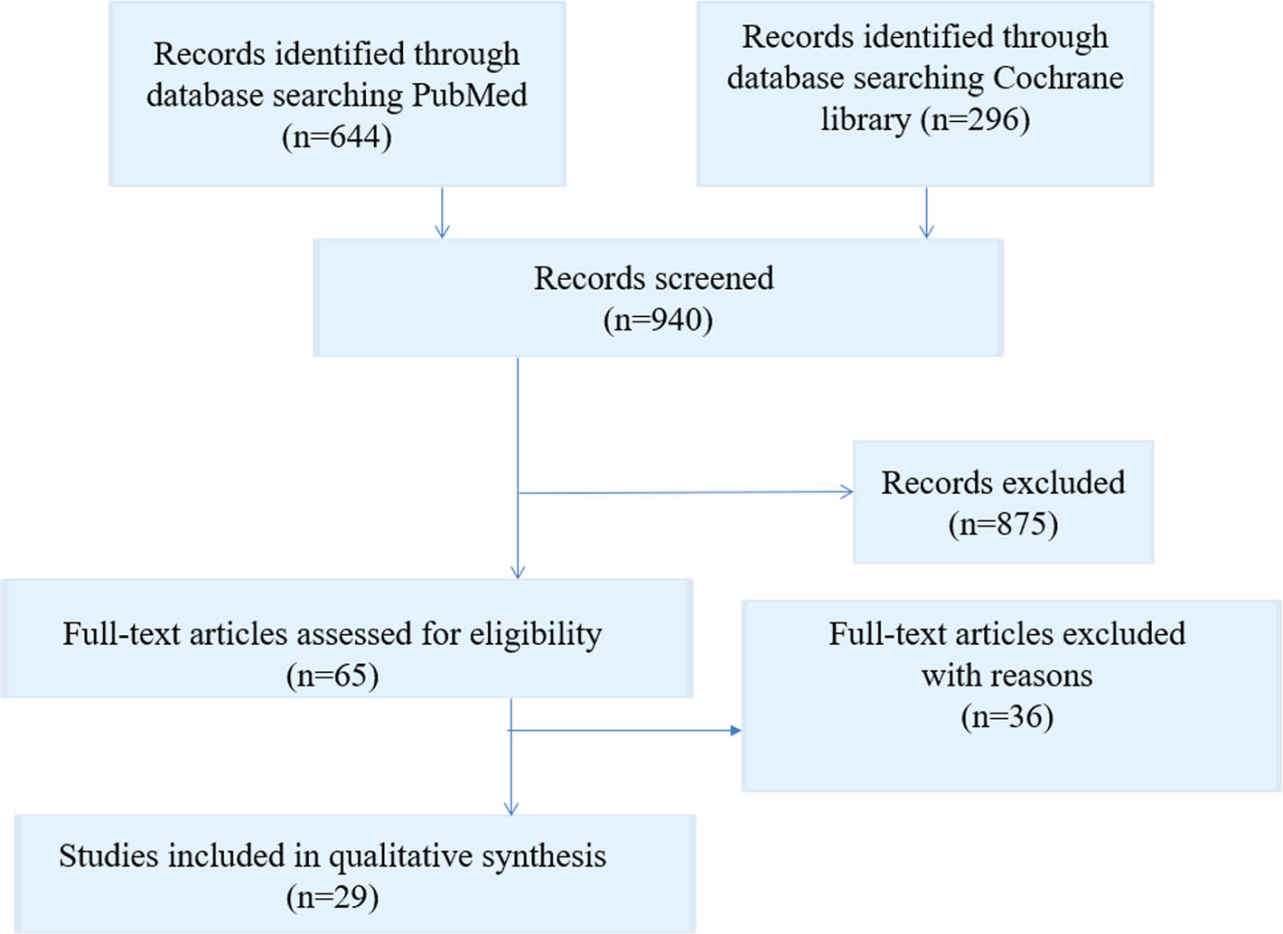
Flow chart literature search strategy

The initial literature search in the two online databases identified 940 records. From these records, 65 full text articles were screened for eligibility. Then 36 full-text articles were excluded because they pertained to patient transfer within the hospital or to another hospital, or to patient hand-over situations. Finally, 29 studies were included in this review. Included studies are listed in Table 2. All document types were searched with a focus on primary research studies. The results of the search strategy are shown in Fig. 1.

**Table 2:** Included studies

Author/ Country/ Year study published/Reference	Study design	Outcome	Source of potential risk to patient safety
Mehta, England (2017) [43] Assessing the impact of the introduction of an electronic hospital discharge system on the completeness and timeliness of discharge communication: A before and after study.	Before and after longitudinal study design, retrospective analysis of discharge summaries for completeness (*N* = 773).	Introduction of a NewEDS (New Electronic Discharge System). Completeness and timeliness of hospital discharge communication.	Risk of delay Risk of lack of information
Maher, Ireland (2013) [50] Use of mobile applications for hospital discharge letters - improving handover at point of practice.	Experimental study (involving fourth-year medical students) (*N* = 80).	Introduction CLAS (Cork Letter-Writing Assessment Scale) checklist; quality of discharge letters written by medical students.	Lack of education
O’Leary, USA (2009) [40] Creating a better discharge summary: improvement in quality and timeliness using an electronic discharge summary.	Survey of medical specialists (outpatient practice), satisfaction with timeliness and quality of summary (*N* = 196).	Presence or absence of 16 components with a summary score for completeness and timeliness, clarity and overall quality using (5-point Likert scales).	Lack of quality Risk of delay
Weiskopf, USA (2013) [66] Sick patients have more data: the non-random completeness of electronic health records.	Comparison of completeness of EHR (electronic health record) and Physical Classification score in randomly selected patients (*N* = 5000).	Relationship between EHR (Electronic Health Record) completeness and patient health status.	Risk of lack of information
Grimes, Ireland (2008) [30] Survey of medication documentation at hospital discharge: Implications for patient safety and continuity of care.	Observational study of cardiology patients admitted over a 3-month period during which a pharmacist prospectively recorded details of medication inconsistencies (*N* = 139).	Discrepancies in medication documentation at discharge.	Risk of lack of information Risk of low quality
Chan, Australia (2014) [41] Improving the efficiency of discharge summary completion by linking to pre-existing patient information databases.	Interventional study. Transfer of electronic data to the discharge summary program improved discharge summary completion rates; reduction in overtime costs (*N* = 10).	1.) Time spent working on discharge summaries. 2.) Time junior medical doctors worked from which hours of overtime was calculated. 3.) Hours of overtime the junior medical doctor claimed. 4.) Proportion of discharge summaries completed within forty-eight hours of patient discharge.	Risk of delay
Lehnbom, Australia (2014) [42] Do electronic discharge summaries contain more complete medication information? A retrospective analysis of paper versus electronic discharge summaries.	Retrospective analysis of paper and electronic discharge summaries (*N* = 199/200).	Completeness of medication information, medication changes during the admission, impact of incomplete information on continuity of care.	Risk of lack of information Risk of low quality in medication information
Bergkvist, Sweden (2009) [37] Improved quality in the hospital discharge summary reduces medication errors-LIMM: Landskrona Integrated Medicines Management.	Longitudinal study with an intervention group and a control group; clinical pharmacists reviewed and gave feedback to the physician on the discharge summary before patient discharge using a structured checklist. Interventional group: (*N* = 52) Control group: (*N* = 63).	Quality of the discharge summary including the medication report and reduction of medication errors in the transition from hospital to primary and community care.	Risk of low quality
Yemm, England (2014) [39] What constitutes a high-quality discharge summary? A comparison between the views of secondary and primary care doctors.	Anonymous survey (*N* = 74) junior doctors at a UK (United Kingdom) general hospital and local GPs (*N* = 153).	Ranking discharge summary key content and characteristics in order of importance (f.e. Accuracy, Completeness, Timeliness, Grammar, Medication changes…).	Risk of low quality Risk of lack of information Risk of delay
Uitvlugt, The Netherlands (2015) [31] Completeness of medication-related information in discharge letters and post-discharge general practitioner overviews.	Observational study (*N* = 99).	Number and percentage of complete medication-related information in the discharge letter and the GP-overview were compared to the TPC- (Transitional Pharmaceutical Care) overview.	Risk of lack of information
Shivji, England (2015) [48] Improving communication with primary care to ensure patient safety post-hospital discharge.	Interventional study, prospective review of electronic discharge summaries over a 6-week period, post-intervention review of discharge summaries, and a further review of discharge summaries was performed after 12 months (*N* = 180 electronic discharge summaries, 60 prospective, 60 post- intervention and 60 after 12 months).	Improvement in discharge summaries and communication with primary care; increasing the content of discharge summaries.	Risk of low quality Risk of lack of education
Cresswell, England (2015) [49] Mind the gap: Improving discharge communication between secondary and primary care.	Interventional study; electronic inpatient discharge documentation (eIDD); documentation of changes to medications and follow-up (*N* = 142).	Implementation of interactive teaching sessions for first year doctors, design of an e-learning module, implementation of new electronic patient record system.	Risk of lack of education Risk of low quality
Ooi, Australia (2017) [32] Improving communication of medication changes using a pharmacist-prepared discharge medication management summary.	Interventional study; retrospective audits of discharge summaries were conducted at baseline and after implementation of the Discharge Medication Management Summary (DMMS) (*N* = 573).	Accuracy of medication change information communicated to GPs; GP satisfaction and feasibility of a pharmacist-prepared Discharge Medication Management Summary (DMMS).	Risk of delay
Belleli, Australia (2013) [27] Communication at the interface between hospitals and primary care: A general practice audit of hospital discharge summaries.	Retrospective study; audit of receipt rates, timeliness and the quality of discharge summaries for 49 admissions in an urban general practice (*N* = 49).	Receipt rates, timeliness and the quality of discharge summaries.	Risk of low quality Risk of lack of information
Wernick, New Zealand (2016) [64] A randomised crossover trial of minimising medical terminology in secondary care correspondence in patients with chronic health conditions: Impact on understanding and patient reported outcomes.	Single-centre, non-blinded, randomised crossover study (*N* = 60 patients).	Minimising the use of medical terminology in medical correspondence→ improved patient understanding and better anxiety/depression scores.	Risk of low patient understanding
Heaton, England (2008) [44] Undergraduate preparation for prescribing: The views of 2413 UK medical students and recent graduates.	Web-based survey; UK medical students and recent graduates about undergraduate training to prescribe and confidence about meeting the relevant competencies (students graduating in 2006–2008 from 25 UK medical schools) (*N* = 2413).	To gather opinions from UK medical students and recent graduates about their undergraduate training to prescribe.	Risk of lack of education
Choudry, USA (2015) [67] Readability of discharge summaries: With what level of information are we dismissing our patients?	Scales [Flesch–Kincaid grade level (FKGL) and Flesch reading ease scores (FRES)] for evaluating readability of medical information (*N* = 497).	Assessment of the health literacy of trauma discharge summaries.	Risk of low patient understanding
Li, Australia (2013) [68] Timeliness in discharge summary dissemination is associated with patients’ clinical outcomes.	Retrospective study on discharge summaries, (*N* = 16.496 patient admissions).	Determination of the relation of readmission of general medical patients to either the existence of a discharge summary or the timeliness of its dispatch.	Risk of delay
Horwitz, USA (2013) [22]Comprehensive quality of discharge summaries at an academic medical center.	Prospective cohort study, patients discharged home after hospitalization for acute coronary syndrome, heart failure, or pneumonia (*N* = 377).	Timeliness of dictation, transmission of the summary to appropriate outpatient clinicians; conduction of a comprehensive quality assessment of discharge summaries.	Risk of delay
Were, USA (2009) [18] Adequacy of hospital discharge summaries in documenting tests with pending results and outpatient follow-up providers.	Retrospective study of a randomly selected sample, patients discharged from two large academic medical centers with pending test results (*N* = 696).	To determine the adequacy with which hospital discharge summaries document tests with pending results and the appropriate follow-up providers.	Risk of delay
Perren, Switzerland (2009) [33] Omitted and unjustified medications in the discharge summary.	Prospective observational review of discharge summaries (*N* = 577).	Evaluation the incidence and types of drug omissions and unjustified medications in the discharge summary; assessment of their potential impact on patient health.	Risk of lack of information Risk of low quality
Tong, Australia (2017) [38] Reducing medication errors in hospital discharge summaries: a randomised controlled trial.	Unblinded, cluster randomised, controlled investigation of medication management plans for patients discharged after an inpatient stay in a general medical unit (Control group *N* = 431) (Intervention group *N* = 401).	Reduction of the rate of medication errors through pharmacists completing medication management plans in the discharge summary.	Risk of low quality
Greer, USA (2016) [26] Hospital discharge communications during care transitions for patients with acute kidney injury: A cross-sectional study.	Cross-sectional review of inpatient hospital medical records (*N* = 75).	To assess the presence and quality of hospital discharge communication about AKI (Acute Kidney Injury).	Risk of low quality Risk of lack of information
Gilmore-Bykovskyi, USA (2018) [19] Hospital discharge documentation of a designated clinician for follow-up care and 30-day outcomes in hip fracture and stroke patients discharged to sub-acute care.	Retrospective cohort study (*N* = 1130).	To assess the relationship between the omission of a responsible clinician/clinic for follow-up care from the hospital discharge summary and poor outcomes for patients transferred to sub-acute care.	Risk of lack of information
Carlsson, Sweden (2012) [20] Accuracy and continuity in discharge information for patients with eating difficulties after stroke.	Prospective, descriptive study (N = 15).	Accuracy and continuity of discharge information for patients with eating difficulties after stroke.	Risk of delay
Walz, USA (2011) [21] Pending laboratory tests and the hospital discharge summary in patients discharged to sub-acute care.	Retrospective cohort study. Stroke, hip fracture, and cancer patients discharged from a single large academic medical center to sub-acute care, 2003–2005 (*N* = 564).	To determine the prevalence and nature of lab tests pending at hospital discharge and their inclusion within hospital discharge summaries for common sub-acute care populations.	Risk of delay
Polyzotis, Canada (2013) [14] Primary care provider receipt of cardiac rehabilitation discharge summaries - are they getting what they want to promote long-term risk reduction?	Cross-sectional study, PCPs (Primary Care Provider) who received a summary were mailed a survey assessing their perceptions of the summaries (N = 577).	To investigate receipt of Cardiac Rehabilitation (CR) discharge summaries by PCPs, as well as timing, and satisfaction with and perceptions of CR summaries.	Risk of delay
Garcia, Norway (2017) [34] Quality of medication information in discharge summaries from hospitals: an audit of electronic patient records.	Randomly selected discharge summaries, evaluation of the medication information (N = 60).	To audit the quality of medication information in discharge summaries and explore factors associated with the quality.	Risk of low quality
Monfort, France (2016) [35] Medication at discharge in an orthopaedic surgical ward: quality of information transmission and implementation of a medication reconciliation form.	Prospective and retrospective study design (*N* = 30).	To assess the completeness of medication information in the medical records, discrepancies between medications noted on the Best Possible Medication at Discharge List (BPMDL) and those prescribed on the discharge order, and the value of the BPMDL for stakeholders.	Risk of lack of information

From these 29 studies, 13 studies dealt with the quality analysis of discharge letters, 12 studies with delayed transmission of medical discharge letters and just as many with the lack of information in medical discharge letters. Only few studies dealt with training on writing medical discharge letters and with understanding of patients of their medical discharge letters. The descriptive information of the included articles is presented in Table 2. Overall quality of the articles was found to be acceptable, with clearly stated research questions and appropriate used methods.

### Risk factors

In the following the identified major risk factors concerning the medical discharge letter are presented in a narrative summary.

#### Delayed delivery

The medical discharge letters should arrive at the GP soon after hospital discharge to ensure the quickest possible further treatment [4]. If letters are delivered weeks after the hospital stay, a continuous treatment of the patient cannot be ensured. Furthermore, the author of the medical discharge letter will no longer have current data after the discharge of the patient, which may result in a loss of important information [10]. Interfaces between different treatment areas and organizational units are known to cause a loss of information and a lack of quality in patient handling [11]. The improvement of information transfer between different healthcare providers during the transition of patients has been recommended to improve patient care [12, 13]. Delayed communication of findings may lead to a lack of continuity of care and suboptimal outcomes, as well as decreased satisfaction levels for both patients and GPs [14–16]. In a review of Kripalani et al., it was shown that 25% of discharge summaries were never received by GPs [17]. This has several negative consequences for patients. Li et al. [18] found that a delayed transmission or absence of the medical discharge summary is related to patient readmission, and a study by Gilmore-Bykovskyi [19] found a strong relationship between patients whose discharge summaries omitted designation of a responsible clinician/clinic for follow-up care and re-hospitalisation and/or death. A Swedish study by Carlsson et al. [20] points out that a lack of accuracy and continuity in discharge information on eating difficulties may increase risk of undernutrition and related complications. A study of Were et al. [18] investigated pending lab results in medical discharge summaries and found that only 16% of tests with pending results were mentioned in the discharge summaries, and Walz et al. [21] found that approximately one third of the sub-acute care patients had pending lab results at discharge, but only 11% of these were documented in the medical discharge summaries.

#### Quality, lack of information

Medical discharge letters are a key communication tool for patient safety issues [17]. Incomplete and insufficient medical discharge letters increase the risks of readmission and myriad other complications [22]. Langelaan et al. (2017) evaluated more than 2000 medical discharge letters and found that in about 60% of the letters essential information was missing, such as a change of the existing medication, laboratory data, and even data on the patients themselves [23]. Accurate and complete medical discharge summaries are essential for patient safety [17, 24, 25]. Addresses; patient data, including duration of stay; diagnoses; procedures; operations; epicrisis and therapy recommendations; as well as findings in the appendix; are minimum requirements that are supposed to be included in the medical discharge letter [4]. However, it was found that key components are often lacking in medical discharge letters, including information about follow-up and management plans [23, 26], test results [27–29], and medication adjustments [30–35]. In a review of Wimsett et al. [36] key components of a high-quality medical discharge summary were identified in 32 studies. These important components were discharge diagnosis, the received treatment, results of investigations as well as follow-up plans.

Accuracy of patients’ medication information is important to ensure patient safety. Hospital doctors expect GPs to continue with the prescribed (or modified) drug therapy. However, the selection of certain drugs is not always transparent for the GPs. A study by Grimes et al. [30] found that a discrepancy in medication documentation at discharge occurred in 10.8% of patients. From these patients nearly 65.5% were affected by discrepancies in medication documentation. The most prevalent inconsistency was drug omission (20.9%). Only 2% of patients were contacted, although general patient harm was assessed. A Swedish study of 2009 [37] investigated the quality improvement of medical discharge summaries. A higher quality of discharge letter led to an average of 45% fewer medication errors per patient.

A recent study by Tong et al. [38] revealed a reduced rate of medication errors in medical discharge summaries that were completed by a hospital pharmacist. Hospital pharmacists play a key role in preparing the discharge medication information transferred to GPs upon patient discharge and should work closely with hospital doctors to ensure accurate medication information that is quickly communicated to GPs at transitions of care [39]. Most hospitals have introduced electronic systems to improve the discharge communication, and many studies found a significant overall improvement in electronic transfer systems due to better documentation of information about follow-up care, pending test results, and information provided to patients and relatives [40–42]. Mehta et al. [43] found that the changeover to a new electronic system resulted in an increased completeness of discharge summaries from 60.7 to 75.0% and significant improvements in levels of completeness in certain categories.

#### Writing of medical discharge letter is missing in medical education

Both junior doctors as well as medical students reported that they received inadequate guidance and training on how to write medical discharge summaries [44, 45] and recognized that higher priority is often given to pressing clinical tasks [46]. Research into the causes of prescribing errors by junior doctors at hospitals in the UK has revealed that latent conditions like organizational processes, busy environments, and medical care for complex patients can lead to medication errors in the medical discharge summary [47].

Fortunately, some study results demonstrate that information and education on writing medical discharge letters would enhance communication to the GPs and prevent errors during the patient discharge process [37]. Minimal formal teaching about writing medical discharge summaries is common in most medical schools [39, 46]; however, a study by Shivji et al. has shown that simple, intensive educational sessions can lead to an improvement in the writing process of medical discharge summaries and communication with primary care [48].

Since the medical discharge letter should meet specific quality criteria, senior physicians and/or the head physician correct(s) and validate(s) the letter. The medical discharge letter therefore represents an essential learning target [8]. Training activities and workshops are necessary for junior doctors to improve writing medical discharge letters [44, 49]. It might be also useful for young doctors to use checklists or other structured procedures to improve writing [4]. Maher et al. showed that the use of a checklist enhanced the quality (content, structure, and clarity) of medical discharge letters written by medical students [50].

In the following Table 3 main risk factors of the medical discharge letter are summarized.

**Table 3:** Major risk factors of the medical discharge letter and improvements to reduce risk factors

Risks factors of the medical discharge letter	Suggestions for improvement
Delayed delivery	Introduction of electronic transfer systems
Quality, lack of information	Training in writing medical discharge letters
Education in writing medical discharge letters	Integration of writing medical discharge letters in study and teaching (checklists, workshops)
Lack of patient understanding	Translation of medical terms, formulation of a (patient-centered) medical discharge letter.

## Discussion

The results of this systematic literature research indicate notable risk factors relating to the medical discharge letter. In a study by Sendlhofer et al., 360 risks were identified in hospital settings [51]. From these, 176 risks were scored as strategic and clustered into “top risks”. Top risks included medication errors, information errors, and lack of communication, among others. During this review, these potential risk factors were also identified in terms of the medical discharge letter.

Delayed sending and low quality of medical discharge letters to the referring physicians, may adversely affect the further course of treatment. However, a study of Spencer et al. has determined rates of failures in processing actions requested in hospital discharge summaries in general practice. It was found that requested medication changes were not made in 17% and patient harm occurred in 8% in relation to failures [52].

Despite the existence of reliable standards [53] many physicians are not adequately trained for writing medical discharge letters during their studies. Regular trainings and workshops and standardized checklists may optimize the quality of the medical discharge letter. Furthermore, electronic discharge letters have the potential to easily and quickly extract important information such as diagnoses, medication, and test results into a structured discharge document, and offer important advantages such as reliability, speed of information transfer, and standardization of content. Comprehensive discharge letters reduce the readmission rate and increase safety and quality by discharging of the patient. A missing structure, as well as a complex language, illegible handwriting, and unknown abbreviations, make reading medical discharge letters more complicated [4]. At least, poor patient understanding of their diagnosis and treatment plans and incomprehensible recommendations can adversely impact clinical outcome following hospital discharge. Many studies confirm that inadequate communication of findings [3, 39, 54] is an important risk factor in patients’ safety [51].

Most medical information in the discharge letter is not understood by patients (as well as relatives and other persons that are involved in the patients’ caring) and patients themselves do not receive a comprehensible medical discharge letter. The content of the medical discharge letter is often useless for the patient due to its medical terminology and content that is not matching with the patient’s level of knowledge or health literacy [55–57]. Poor understanding of diagnoses and related discharge plans are common among patients and family members and often accompanied by unplanned hospital readmissions [58–61]. In a study by Lin et al., it was shown that a patient-directed discharge letter enhanced understanding for hospitalization and for recommendations. Furthermore, verbal communication of the letter contents, explanation of every section of the medical discharge letter, and the opportunity for discussion and asking questions improved patient comprehension [7]. A study by O’Leary et al. showed that roughly 80–95% of patients with breast tumours want to be informed and educated about their illness, treatment, and prognosis [62].

High quality of care is characterized by a patient-centered communication, where the patient’s personal needs are also in focus [63]. Translation of medical terms in reports and letters leads to a better understanding of the disease and, interestingly, the avoidance of medical terms did not lead to deterioration in the transmission of information between the treating physicians. Moreover, it was found that the minimisation of medical terminology in medical discharge letters improved understanding and perception of patients’ ability to manage chronic health conditions [64]. In effect, it is clear that patient-centered communication improves outcome, mental health, patient satisfaction and reduces the use of health services [65].

## Strengths and limitations

We have identified key problems with the medical discharge summaries that negatively impact patients’ safety and wellbeing. However, there is a heterogeneous nature of the included studies in terms of study design, sample size, outcomes, and language. Only two reviewers screened the studies for eligibility and only full-text articles were included in the literature review; furthermore, only the databases Pubmed and Cochrane library were screened for appropriate studies. Due to these constraints, there is a chance that other relevant studies may have been missed.

## Conclusions

High-quality medical discharge letters are essential to ensure patient safety. To address this, the current review identified the major risk factors as delayed sending and low quality of medical discharge letters, lack of information and patient understanding, and inadequate training in writing medical discharge letters. In future, research studies should focus on improving the communication of pending test results and findings at discharge, and on evaluating the impact that this improved communication has on patient outcomes. Moreover, a simple patient-centered medical discharge letter may improve the patient’s (as well as family members’ and other caregivers’) understanding of disease, treatment and post-discharge recommendations.

## Abbreviations

GP: General practitioner
RCT: Randomized Controlled Trial
STROBE: STrengthening the Reporting of OBservational studies in Epidemiology
UK: United Kingdom
